# Vaspin antagonizes high fat-induced bone loss in rats and promotes osteoblastic differentiation in primary rat osteoblasts through Smad-Runx2 signaling pathway

**DOI:** 10.1186/s12986-020-0429-5

**Published:** 2020-01-22

**Authors:** Hongwei Wang, Fulian Chen, Jiaxuan Li, Yan Wang, Chunyan Jiang, Yan Wang, Mengqi Zhang, Jin Xu

**Affiliations:** 1 Department of Endocrinology, Shandong Provincial Hospital affiliated to Shandong University, Jinan, Shandong People’s Republic of China; 2 Shandong Provincial Key Laboratory of Endocrinology and Lipid Metabolism, Jinan, Shandong People’s Republic of China; 3 Institute of Endocrinology and Metabolism, Shandong Academy of Clinical Medicine, Jinan, Shandong 250021 People’s Republic of China; 4 Department of Clinical Nutrition, People’s Hospital of Rizhao, Rizhao, Shandong 276800 People’s Republic of China; 5 Department of Endocrinology, Affiliated Yidu Central Hospital of Weifang Medical College, Weifang, Shandong 272500 People’s Republic of China; 6 Department of Endocrinology, The Second Affiliated Hospital of Shandong First Medical University, Tai’an, Shandong 271000 People’s Republic of China; 7 Department of Endocrinology, People’s Hospital of Linyi, Linyi, Shandong 276000 People’s Republic of China

**Keywords:** Vaspin, High fat diet, Osteogenic differentiation, Smad2/3, P-Smad2/3, Runx2

## Abstract

**Background:**

Visceral adipose tissue-derived serine protease inhibitor (vaspin), an adipose-derived hormone, exhibits various biological functions. Recently, studies showed that vaspin is closely related to bone metabolism. However, how vaspin influences bone formation and its underlying mechanisms in high fat-induced obese rats and rat primary osteoblasts (OBs) are not fully understood. In this study, the effects of vaspin on bone mechanical parameters and microarchitecture were evaluated.

**Methods:**

A total of 40 male Sprague-Dawley (SD) rats at 5-week old were fed with high fat diet (HFD) and normal diet (ND) for 12 weeks followed by treatment of vaspin for 10 weeks. Micro CT and three-point bending tests were conducted to evaluate bone microstructure and biomechanics. The alkaline phosphatase (ALP) activity, expression of Runt-related transcription factor 2 (Runx2), Osterix (Osx), Collegen alpha1 (Colla1) procollagen I N-terminal peptide (PINP), C-telopeptide of type I collagen (CTX), Smad2/3 and p-Smad2/3 was detected by different methods.

**Results:**

Our data indicated that, compared with ND rats, HFD rats exhibited high body weight, decreased bone strength and deteriorative bone quality. In contrast, vaspin reduced the body weight, improved the whole body metabolic status, enhanced bone strength, trabecular bone mass, and expression of Runx2, Osx, PINP, and decreased the expression level of plasma CTX. In vitro studies showed that vaspin promoted osteogenic differentiation and ALP activity in rat primary OBs in a dose dependent manner. Vaspin also upregulated mRNA expression of osteogenesis-related genes Runx2, Osx and Colla1 and protein expression of Runx2, Smad2/3 and p-Smad2/3.

**Conclusions:**

Our results indicated that vaspin protects against HFD-induced bone loss, and promotes osteogenic differentiation by activating the Smad2/3-Runx2 signaling pathway*.*

## Introduction

Osteoporosis and obesity are interrelated metabolic derangements, which are serious and prevalent health issues [1]. Osteoporosis and related bone fractures are growing medical problems affecting more than 200 millions of people worldwide and appear to be associated with high disability and mortality, especially in older men and postmenopausal women [2]. Obesity is widely recognized as one of the most serious risk factors for chronic diseases including insulin resistance, metabolic syndrome, type 2 diabetes mellitus, cardiovascular complications and cancers [3]. Traditionally, evidence suggests that obesity protects against osteoporosis [4]. Nevertheless, emerging findings suggest that excess fat mass is a risk factor for bone loss in human [5]. Lac et al. [6] demonstrated that high fat diet (HFD) intake during the growing period has deleterious effects on bone parameters in rats. Burchfield et al. [7] found that prolonged exposure to HFD results in morbid obesity and led to extensive bone loss in mice. Other studies also showed that HFD-induced obesity (DIO) increases bone resorption and/or decrease bone formation, resulting in reduced bone mass and bone strength in various rodent models [8]. So far, obesity is reported to affect bone metabolism through several potential mechanisms. For instance, obesity tends to be accompanied by excessive consumption of HFD, and related to a chronic inflammation condition characterized by the increased plasma levels of proinflammatory cytokines such as tumor necrosis factor α (TNF-α), interleukin-6 (IL-6), and interleukin-1 (IL-1). These cytokines are known to stimulate the proliferation and differentiation of osteoclasts and might enhance bone resorption [9]. Adipocytes and osteoblasts are derived from common multipotential mesenchymal stem cells, obesity increases bone marrow adipogenesis while inhibits osteoblastogenesis. Furthermore, obesity is usually accompanied with abnormal secretion of adipokines-adiponectin, leptin, ghrelin, and resistin, which may affect the bone mineral density (BMD) through different pathways such as transforming growth factor-β (TGF-β) signaling, the Receptor activator of nuclear factor kappa-Β ligand (RANKL)/RANK/osteoprotegerin (OPG) pathway, and the Peroxisome proliferator-activated receptor gamma (PPAR-γ) pathway [10]. Previous studies demonstrated that administration of leptin prevents bone loss in ovariectomized rats [11], promotes bone formation in ob/ob mice [12], indicating a positive effect on the progress of fracture healing in SD rats [13] and adiponectin treatment increases trabecular bone mass [14].

As a newly discovered adipokine, visceral adipose tissue-derived serine protease inhibitor (vaspin) was identified as a member of the serine protease inhibitor (serpin) family, which is highly expressed in visceral adipose tissue when obesity and insulin levels peak in Otsuka Long-Evans Tokushima Fatty (OLETF) rats [15]. To present, the researchers and their teams mainly focused on the influences of vaspin on insulin resistance [16], hepatitis disease [17], and cardiovascular disease [18]. Administration of vaspin in obese mice and rats improves glucose tolerance, insulin sensitivity and reduces food intake [19, 20].

Notably, emerging studies have found that vaspin is closely related to bone metabolism in vitro. Recent data showed that vaspin attenuates RANKL-induced osteoclast formation in RAW 264.7 cells, decreases the apoptosis of human osteoblasts, and regulates the osteogenic differentiation of MC3T3-E1 [21, 22]. Therefore, it is logical to hypothesize that vaspin exerts a positive effect on bone metabolism. However, the effects and mechanisms of vaspin on bone metabolism in vivo remain unknown. In this study, we thus aimed to clarify the biological roles of vaspin in the HFD-induced bone loss and to explore the relationship between vaspin and the Smad-Runx2 signaling pathway in vitro for revealing the new mechanism of vaspin functions.

## Materials and methods

### Animal model and vaspin treatment

The study protocol was reviewed and approved by the Animal Care and Use Committee at Shandong Provincial Hospital (Jinan, China). Forty male Sprague-Dawley (SD) rats (Vital River Corporation, Beijing, China) at age of 4 weeks old were housed under 12-h light/dark cycle with 50% humidity at 23 °C conditions, and they freely accessed to food and water. After one week acclimation, the rats were randomly separated into two groups: rats fed with normal diet (ND, *n* = 20) or high fat diet (HFD, n = 20, containing 60%kCal%fat, D12492, Research Diets, New Brunswick, NJ, USA) for 12 weeks to develop the control group and DIO group. Then the control group was randomly divided into two subgroups: ND (*n* = 10), ND + vaspin (n = 10). The DIO group was also randomly divided into two subgroups: HFD (n = 10) and HFD + vaspin (n = 10). During the following 10 weeks of the study, ND + vaspin rats and HFD + vaspin rats were treated with subcutaneous injection of 1 μg/kg body weight recombinant vaspin once a day (Novoprotein Co., Ltd., Shanghai, China, C192) or saline according to a previous report [19]. Food intake and body weight were monitored weekly. At the end of the experiments, blood samples and femurs were collected after euthanizing the rats. The femurs were collected from the sacrificed animals and cleaned of soft tissue. The right femurs were packed in gauze soaked with a NaCl solution (9 g/L) and stored at − 20 °C, while the left femurs were fixed with 75% ethanol and stored at 4 °C for the following experiments.

### Assessment of body metabolic status using metabolic chambers

After 10 weeks of vaspin treatment, rats were acclimated in individual metabolic chambers (PhenoMaster, TSE Systems, Germany) with a 12 h dark-light cycle at 24 °C for 48 h. Measurement indicators included volume of oxygen consumed (VO_2_), production of carbon dioxide (VCO_2_), respiratory exchange ratio (RER), heat production (H) and physical activity.

### Serum biochemical analysis

Serum calcium (Ca), inorganic phosphorus (P), total cholesterol (TC), triacylglycerol (TG), blood glucose, high-density lipoprotein cholesterol (HDL) and low-density lipoprotein cholesterol (LDL) concentrations were measured by automatic biochemical analyzer (Olympus Co., Ltd., Tokyo, Japan). In addition, serum concentrations of a bone formation marker procollagen I N-terminal peptide (PINP), and a bone resorption marker C-telopeptide of type I collagen (CTX) were measured with ELISA kits according to the protocols supplied by the manufacturer (Cusabio, Wuhan, China).

### Bone biomechanical analysis

A three-point bending test was performed on ElectroForce dynamic mechanical testing system (BoseElectroForce®3230, America) to assess bone strength. The bone biomechanical indexes were calculated as described in previous study [23]. The right femurs in the ice slowly unfrozen at room temperature and were kept humidity. Then the bones were placed in instrument on the femoral level, make the surface downward, on two supports that were equidistant from the ends and 8 mm apart and the length of the femurs were measured with vernier caliper. The load measurement precision was 0.01 N, and displacement measurement accuracy was 0.001 mm. The load was applied at a constant deformation rate of 2 mm/min. The diaphysis of the femurs was loaded until a fracture occurred to determine the yield and fracture parameters. The yield represented the point at which bone ceases to behave elastically. The data were automatically recorded in a computer interfaced to the testing machine, and a typical load-deformation curve was created. The maximum load, maximum fracture load, stiffness, energy absorption, elastic modulus, and the maximum strength were measured.

### Microcomputed tomography analysis

Microcomputed tomography (Micro-CT) was performed using a Skyscan 1176 μCT scanner (Bruker, Belgium) with a slight modification from previous reports [24, 25]. The left femurs were scanned with the following parameters: scanning accuracy was 17.93 μm, 180° rotating scanning, filter for 1 mm A1, scanning voltage 70kv, current 278 μA, and exposure time 450 ms. The cortical bone parameters including volume bone mineral density (Ct.vBMD), bone volume/total volume (Ct.BV/TV), and cortical thickness (Ct.Th). The trabecular bone parameters including trabecular volume bone mineral density (Tb.vBMD), trabecular bone volume/total volume (Tb.BV/TV), trabecular number (Tb.N), trabecular thickness (Tb.Th), trabecular separation (Tb.Sp) and the structure model index (SMI) were obtained. All data were generated using CT-Analyzer software (Skyscan).

### Cell culture

Rat primary OBs were isolated from the calvarias of newborn baby SD rats within 72 h by sequential enzymatic digestion, as described previously [26, 27]. In brief, the calvarias, consisting of frontal and parietal bones, were dissected aseptically, and subjected to consecutive digestions at 37 °C in Hefley’s buffer containing 0.1% type II collagenase and 0.25% trypsin. Cells and debris released during the first two, 20-min digestions, were discarded and cells obtained from the third, 60-min digestions were plated in α-minimum essential medium (MEM) supplemented with 10% fetal bovine serum (FBS), 2 mM L-glutamine (Sigma-Aldrich, USA), 100 U/mL penicillin-streptomycin (Sigma-Aldrich, St. Louis, Missouri, USA) in a humidified 5% CO2 incubator at 37 °C. The culture medium was changed every 2 days. Cells were grown for several days until reaching 90% confluence, and were then trypsinized and plated in the same medium for subsequent experimental procedures. All primary osteoblasts used in experiments were between 2th and 5th passage.

### Cell proliferation assay

To assess the effects of vaspin on cell proliferation, cells were seeded in 96-well plates. After 12 h, 24 h, 48 h, 72 h incubation with different concentrations of vaspin (10 ng/ml, 50 ng/ml, 100 ng/ml), based on previous study [21], cells were respectively treated with 10% cell counting kit-8 (CCK-8, Dojindo, DJ657, Japan) in 150 mL a-MEM without FBS for 3 h at 37 °C. Absorbance was measured at 450 nm on a microplate reader (spectra max plus 384, Jinan Dongdai Scientific Equipmet Co.,Ltd., Jinan, China). Cell viability (%) = [OD (vaspin)-OD (blank)]/[OD (control)-OD (blank)] × 100.

### Alkaline phosphatase assay

For the alkaline phosphatase (ALP) staining assay, OBs were seeded into 96-well plates and incubated with vaspin at various concentrations (10 ng/ml, 50 ng/ml, 100 ng/ml) for 72 h respectively. Intracellular ALP activity was measured using an ALP assay kit (Jiancheng Biotechnology, Institute, Nanjing, China) as previously described [27]. The absorbance was measured at a wavelength of 520 nm according to the manufacturer’s protocol.

### RNA extraction and real-time quantitative RT-PCR

Total RNA was isolated from distal metaphyses of the right femurs and cultured osteoblasts using RNAisoTMPlus (TakaRa, Dalian, China), and then reverse-transcribed into single-stranded cDNA using Prime Script®RT reagent kit (TakaRa, Dalian, China). Quantitative real-time PCRs of Runt-related transcription factor 2 (Runx2), Osterix (Osx), Collegen alpha1 (Colla1) and β-actin were performed on an equal amount of cDNA using SYBR®Premix Ex TaqTMII according to the instructions of the manufacturer (TakaRa, Dalian, China). The primers used were as follows in Table 1. Real-time PCR was performed on the LightCycler480 (Roche Diagnostics, Mannheim, Germany) with an initial denaturation step of 30 s at 95 °C, followed by 40 cycles of 5 s at 95 °C, and 20s at an annealing temperature at 60 °C. The relative mRNA expression levels were normalized to the β-actin in the same sample. The Data analysis was performed with the 2^-△△CT^ method.

**Table 1:** Primer sequences used for the determination of gene expression

Gene (Rats)	Primer sequence((5′ - 3′)
Colla1	Forward GCGAAGGCAACAGTCGCT
	Reverse CTTGGTGGTTTTGTATTCGATGAC
Runx2	Forward CCTGAACTCAGCACCAAGTCCT
	Reverse TCAGAGGTGGCAGTGTCATCA
Osx	Forward CTGGGAAAAGGAGGCACAAAGA
	Reverse GGGGAAAGGGTGGGTAGTCATT
β-actin	Forward TGCTATGTTGCCCTAGACTTCG
	Reverse GTTGGCATAGAGGTCTTTACGG

### Western blot analysis

Total cellular protein was prepared using a standard method [28]. After different concentrations of vaspin were used to stimulate cells for 48 h, the osteoblasts were rinsed with ice-cold PBS and lysed in radio-immunoprecipitation assay (RIPA) lysis buffer (Shenergy Biocolor Bioscience & technology CO., Shanghai, China) for obtaining total protein according to the manufacturer’s instructions. The protein concentration was measured by the bovine serum albumin (BCA) Protein Quantitative Assay Kit (Shenergy Biocolor Bioscience & technology CO., Shanghai, China). Proteins were separated using sodium dodecyl sulfate–polyacrylamide gel electrophoresis (SDS-PAGE) with 10% polyacrylamide gels, transferred to a polyvinylidene difluoride (PVDF) membrane (Millipore, Billerica, MA, USA) and blotted with specific antibodies including anti-phospho-Smad2 (1:1000, Cell Signaling Technology, Danvers, MA, USA), anti-Smad2 (1:1000, Cell Signaling Technology, Danvers, MA, USA), anti-phospho-Smad3 (1:1000, Cell Signaling Technology, Danvers, MA, USA), anti-Smad3 (1:1000, Cell Signaling Technology, Danvers, MA, USA), anti-Osx (1:1000, Abcam, Cambridge, MA, USA), anti-Runx2 (1:1000, Abcam, Cambridge, MA, USA), anti-β-catenin (1:1000, Abcam, Cambridge, MA, USA) and anti-GAPDH (1:1000, Abcam, Cambridge, MA, USA). Protein bands following incubation with an appropriate secondary antibody were detected. The relative target protein levels were normalized to GAPDH in the same membrane.

### Immunofluorescence staining

Primary rat osteoblasts were grown on glass coverslips and incubated with vaspin for 72 h. The cells were washed with cold PBS twice, fixed in ice-cold methanol and permeabilized with 0.5% Triton X-100 for 15 min. BSA was used to block nonspecific binding sites. Samples were then incubated with anti-Runx2 antibody (1:500, Abcam, Cambridge, MA, USA) overnight at 4 °C followed by incubation with tetramethylrhodamine (TRITC)-conjugated secondary antibody and 4′,6-diamidino-2-phenylindole (DAPI). The samples were mounted and observed under a confocal fluorescence microscopy (Olympus, Tokyo, Japan).

### Statistical analysis

Results were expressed as means ± standard deviation (SD). Data were analyzed by two-tailed unpaired Student’s t test for two groups and one-way analysis of variance (ANOVA) test for multiple groups, using Statistical Package for the Social Sciences (SPSS) software version 17.0 (SPSS, Inc., Chicago, IL, USA). The value of *p* < 0.05 was considered significantly.

## Results

### Effects of vaspin on the body weight and food intake of SD rats

**Fig. 1:**
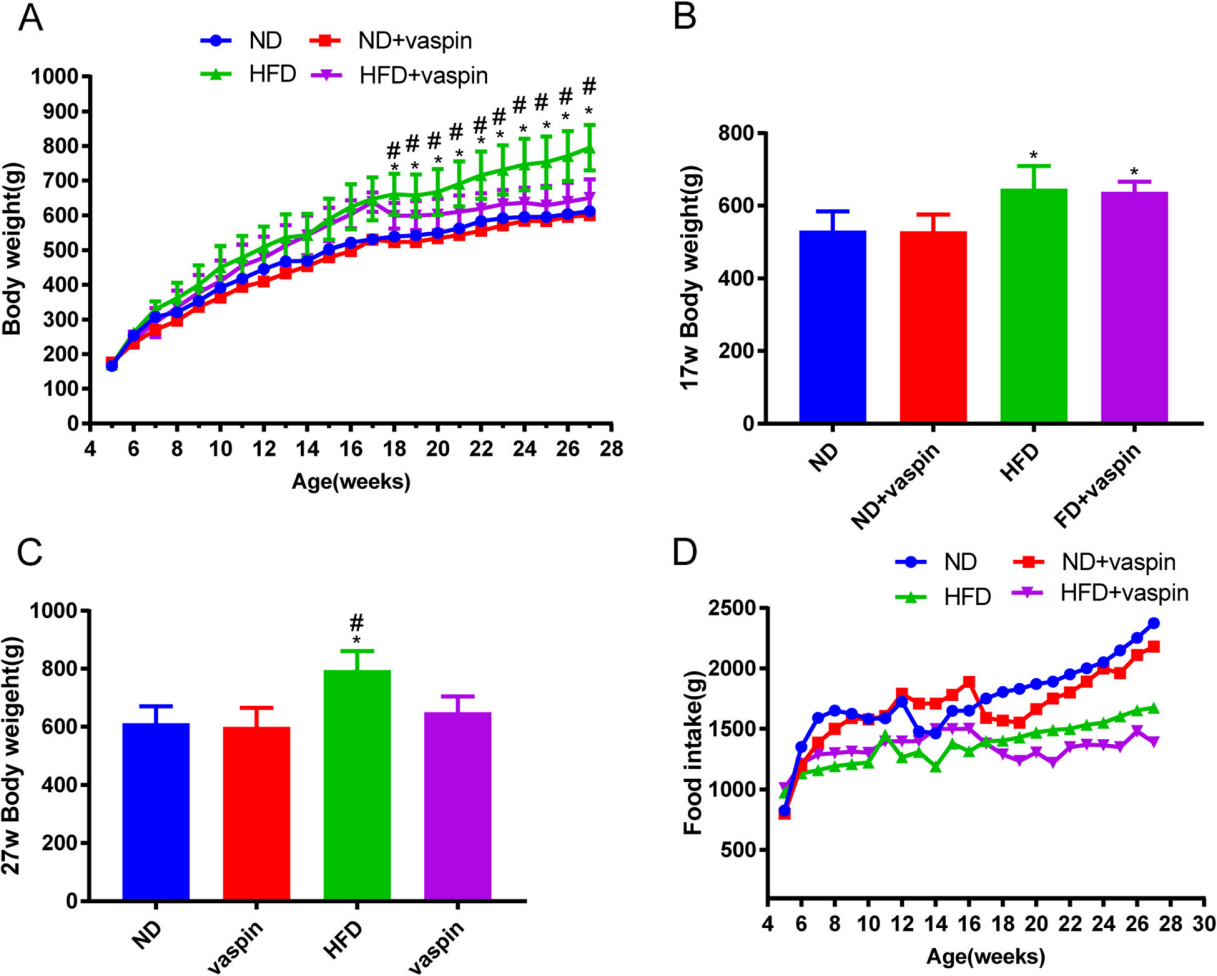
Effects of vaspin on the body weight and food intake of SD rats. Body weight was detected weekly during rats were fed with high fat diet or normal diet (**a**). Body weight of rats fed with high fat diet or normal diet for 12 weeks (**b**). Body weight of rats after vaspin treatment for another 10 weeks (**c**). Comparisons of food intake at different ages (**d**). Data are expressed as mean ± SD (*n* = 10). ^*^p < 0.05 compared with ND, ^#^*p* < 0.05 compared with HFD + vaspin

Body weight was detected weekly shown as the weight gain curve (Fig. 1a). After rats were fed with high fat for 12 weeks, the body weight of HFD group was significantly higher than that of ND group (*P* < 0.05, Fig. 1b). However, after vaspin intervention for 10 weeks, the final body weight of HFD + vaspin group was significantly reduced compared to the HFD group (*P* < 0.05, Fig. 1c). There was no difference in body weight among the ND, ND + vaspin and HFD + vaspin group (*P* > 0.05, Fig. 1c). On the other hand, in the tested vaspin dose, neither the ND + vaspin group nor the HFD + vaspin group showed any significant difference on food intake, compared to the ND group and the HFD group respectively (Fig. 1d). Taken together, these data indicated that the administration of vaspin for 10 weeks can significantly decrease the body weight gain in HFD-fed rats, while it shows no significant effect on the food intake.

### Effects of vaspin on the whole body metabolic status

**Fig. 2:**
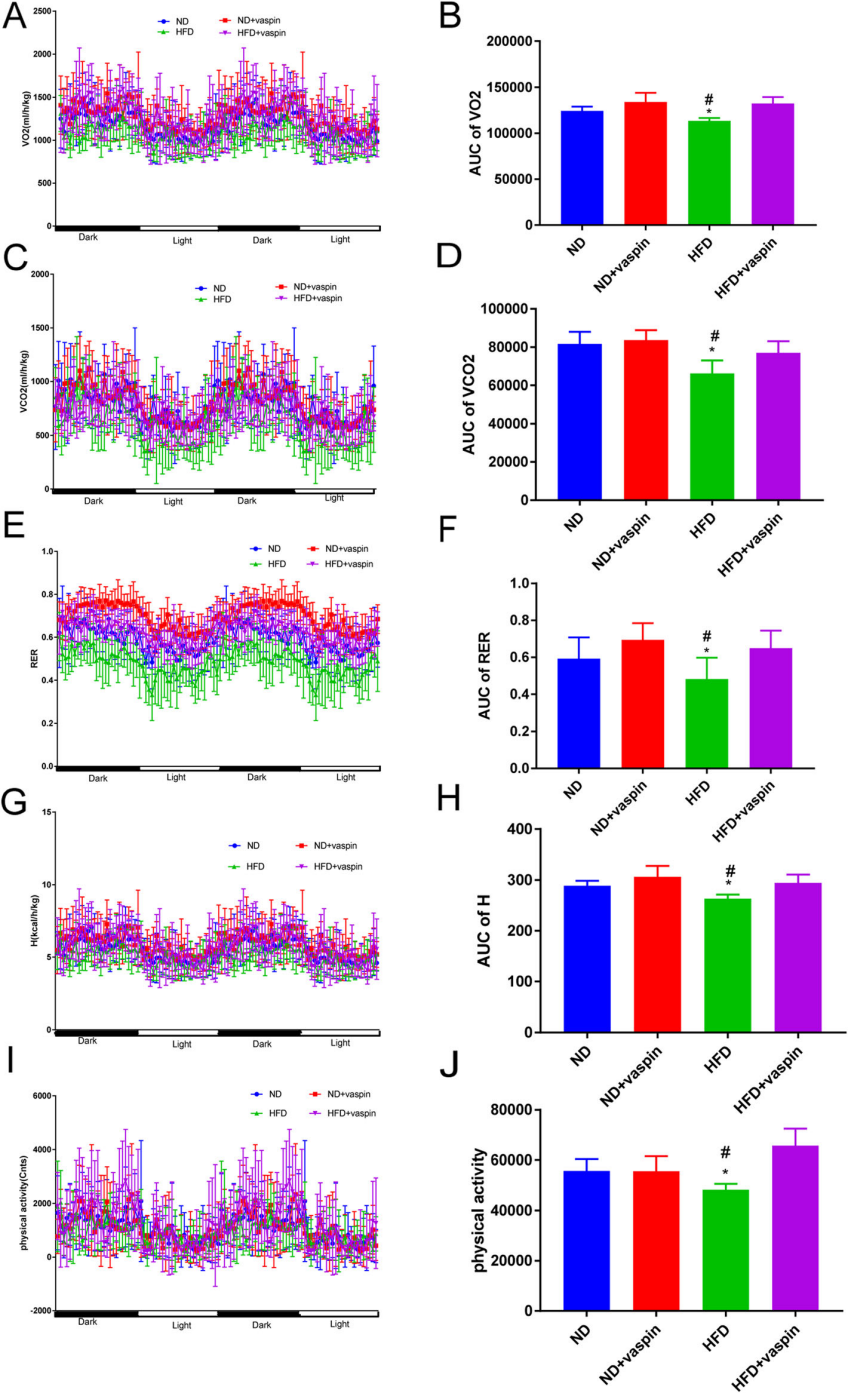
Effects of vaspin on the whole body metabolic status. After 10 weeks of vaspin treatment, the rats (*n* = 8) were placed into metabolic chambers to measure the VO_2_, VCO_2_, RER and other parameters in different groups. The VO_2_ (**a**), AUC for VO_2_ (**b**), VCO_2_ (**c**), AUC for VCO_2_ (**d**), RER (**e**), AUC for RER (**f**), heat production (**g**), AUC for heat production (**h**), physical activity (**i**), and AUC for physical activity (**j**) was recorded. ^*^p < 0.05 compared with ND, ^#^*p* < 0.05 compared with HFD + vaspin. Data are expressed as mean ± SD

As shown in Fig. 2, the VO_2_, VCO_2_, RER, H and physical activity were reduced in the HFD rats as compared to the ND rats. The area under the curve (AUC) for the above parameters also supported the conclusion. Above results suggested that the whole body metabolism of rats was significantly decreased in the HFD rats in comparison to the ND rats, while vaspin can improve the whole body metabolic parameters.

### Effects of vaspin on serum biochemical markers

**Fig. 3:**
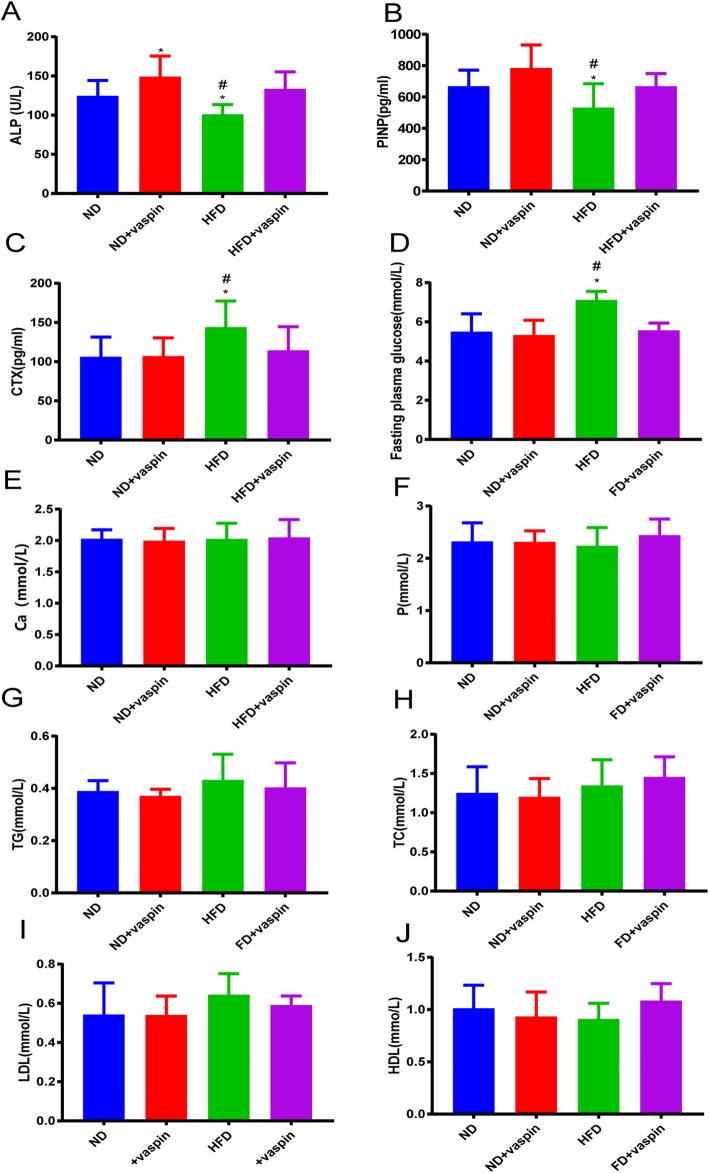
Effects of vaspin on serum biochemical markers. Serum concentrations of ALP (**a**), PINP (**b**), and CTX (**c**) were measured. The changes in fasting plasma glucose in different groups were determined (**d**). The serum levels of Ca, P, TG, TC, LDL, HDL in different groups (**e**-**j**). Data are expressed as mean ± SD (n = 8).^*^p < 0.05 compared with ND, ^#^*p* < 0.05 compared with HFD + vaspin

Results of vaspin on serum biochemical markers were shown in Fig. 3. The serum ALP level of ND + vaspin group was significantly increased compared to ND group, but it was significantly decreased in HFD group compared to ND, ND + vaspin, and HFD + vaspin groups (Fig. 3a). There was no difference in the serum ALP level between the ND and HFD + vaspin group. Serum concentrations of PINP and CTX were also measured, and found that the serum PINP level was significantly lower in rats in the HFD group than that in the ND, ND + vaspin, and HFD + vaspin groups, while there were no difference in the serum PINP level between the ND, ND + vaspin, and HFD + vaspin groups (Fig. 3b). On the other hand, CTX expression was significantly promoted in the HFD-treated rats compared to the ND, ND + vaspin, and HFD + vaspin-treated rats (Fig. 3c), suggesting that high fat feeding suppresses bone formation, while vaspin supplementation can antagonize the effect of HFD. The serum levels of Ca, P, TG, TC, LDL, HDL were not affected either by HFD or vaspin supplementation.

### Effects of vaspin on bone biomechanics

**Fig. 4:**
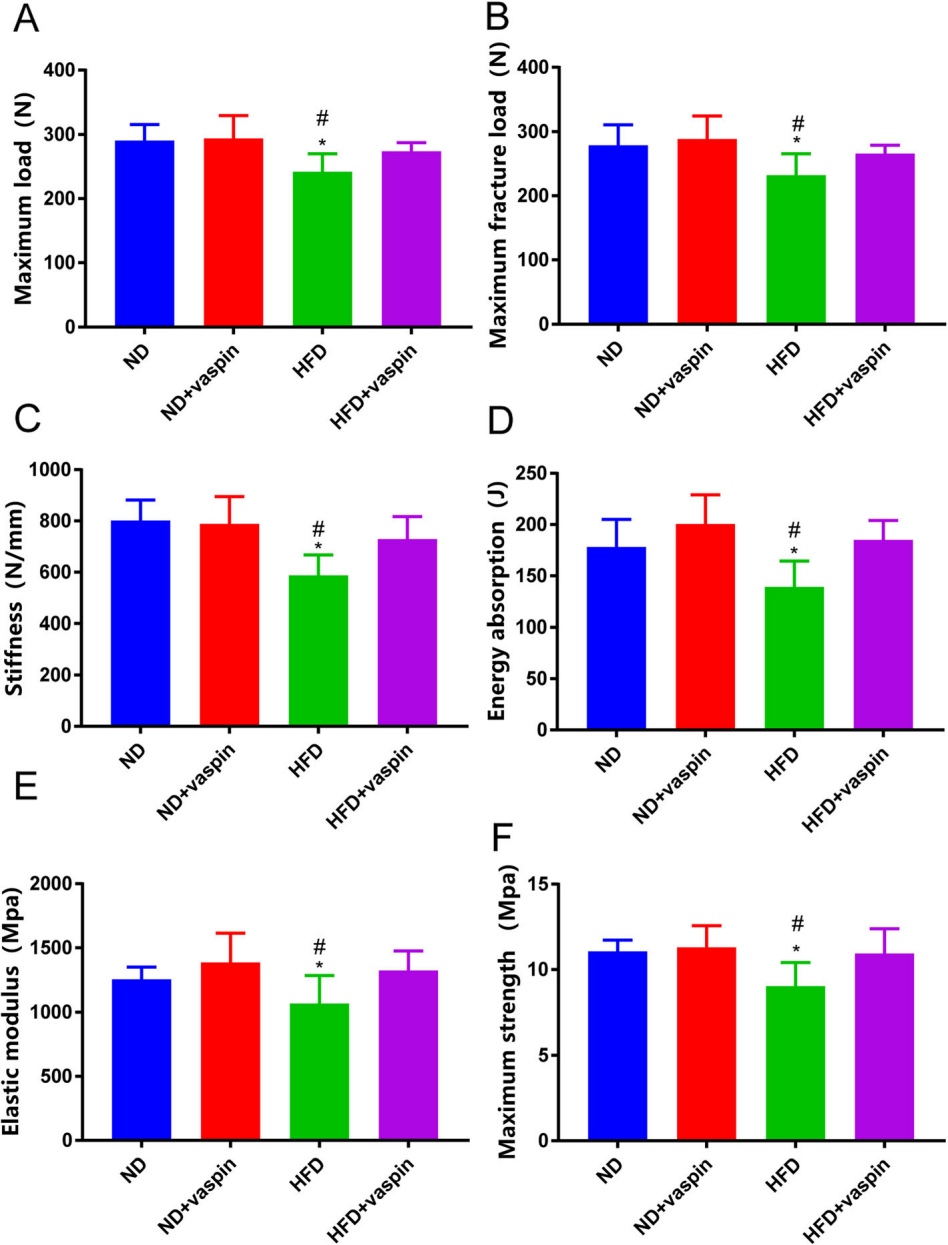
Effects of vaspin on bone biomechanics. Maximum load (**a**), maximum fracture load (**b**), stiffness (**c**), energy absorption (**d**) elastic modulus (**e**) and maximum strength (**f**) were detected following treatment of vaspin. Data are expressed as mean ± SD (n = 8). ^*^p < 0.05 compared with ND, ^#^*p* < 0.05 compared with HFD + vaspin

Bone strength was determined by biomechanical tests, and the results were shown in Fig. 4. The maximum load, maximum fracture load, stiffness, energy absorption, elastic modulus, and the maximum strength of the HFD group were significantly decreased compared to ND, ND + vaspin, HFD + vaspin groups. However, there were no differences among the ND, ND + vaspin, HFD + vaspin groups. The results indicate that bone strength is significantly reduced in HFD rats relative to ND rats, while vaspin treatment can improve bone strength-related parameters.

### Effects of vaspin on microcomputed tomography

**Fig. 5:**
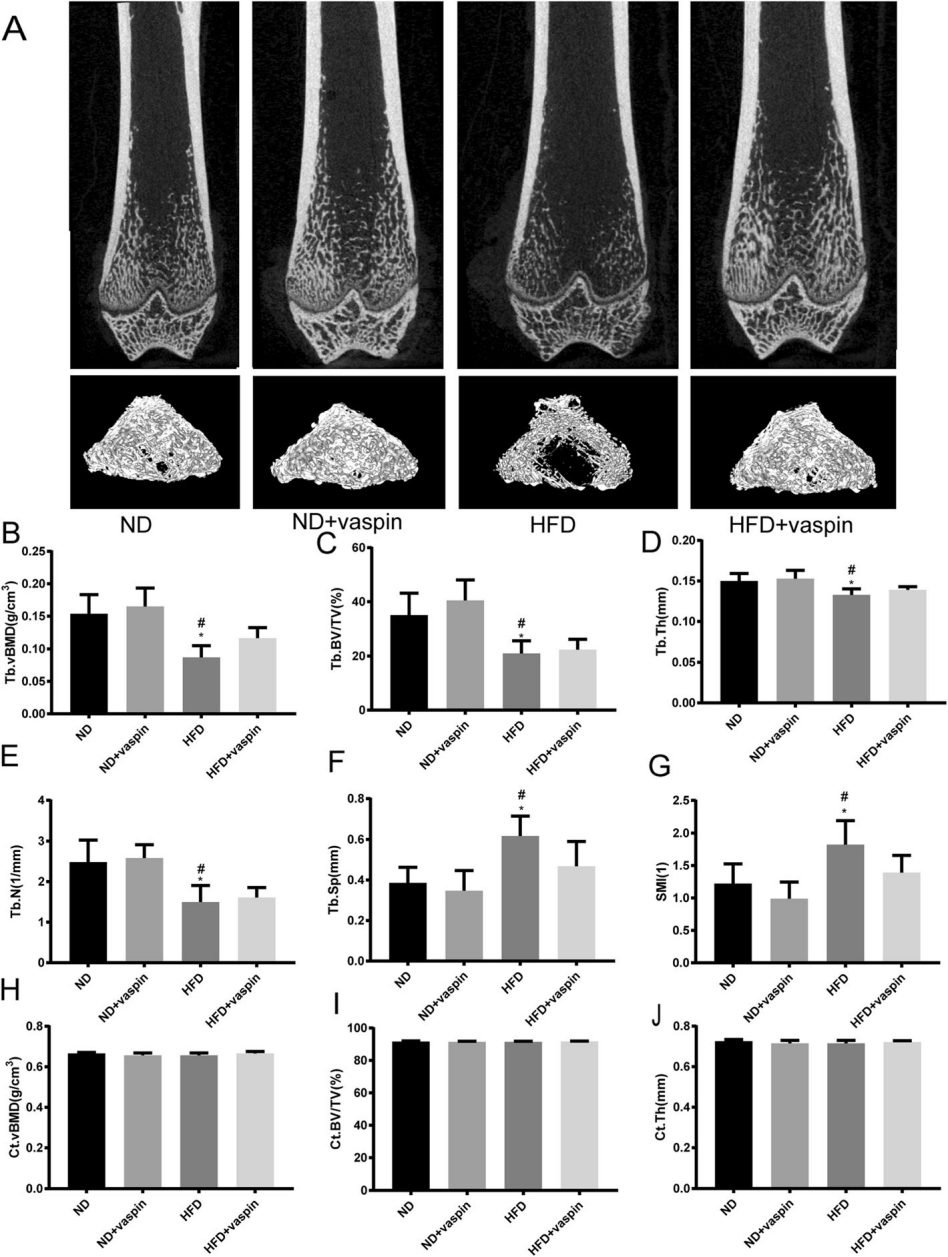
Effects of vaspin on microcomputed tomography. 2D-microCT images and 3D-microCT images of femurs in different groups (**a**). Tb.vBMD, trabecular volume bone mineral density; Tb.BV/TV, trabecular bone volume/total volume; Tb.Th, trabecular thickness; Tb.N, trabecular number; Tb.Sp, trabecular separation and SMI, the structure model index (**b**-**g**). Ct.vBMD, cortical volume bone mineral density; Ct. BV/TV, cortical bone volume/total volume; Ct. Th, cortical bone thickness (**h**-**j**). ^*^p < 0.05 compared with ND, ^#^*p* < 0.05 compared with HFD + vaspin. Data are expressed as mean ± SD (n = 8)

Micro-CT was used to evaluate effects of vaspin and HFD on microstructural properties of femurs, the results were shown in Fig. 5. The positive effects of vaspin on trabecular bone mass and microarchitecture deterioration in HFD-induced obese rats were further supported by 2D-microCT images and 3D-microCT images (Fig. 5a). Compared with the ND group, HFD diet caused a significant decrease in trabecular bone parameters including Tb.vBMD, Tb.N, Tb.Th, and Tb.BV/TV, and an increase in Tb.Sp and SMI. Conversely, 10 weeks of vaspin intervention prevented HFD-induced bone loss and microarchitecture deterioration, as evidenced by the increased Tb.vBMD, Tb.N, Tb.Th, and Tb.BV/TV, the decreased Tb.Sp and SMI compared with the HFD group (Fig. 5b-g). Furthermore, neither 12 weeks of HFD nor 10 weeks of vaspin intervention had an impact on cortical bone parameters including Ct.vBMD, Ct.BV/TV, and Ct.Th (Fig. 5h-j).

### Effects of vaspin on osteoblast proliferation

**Fig. 6:**
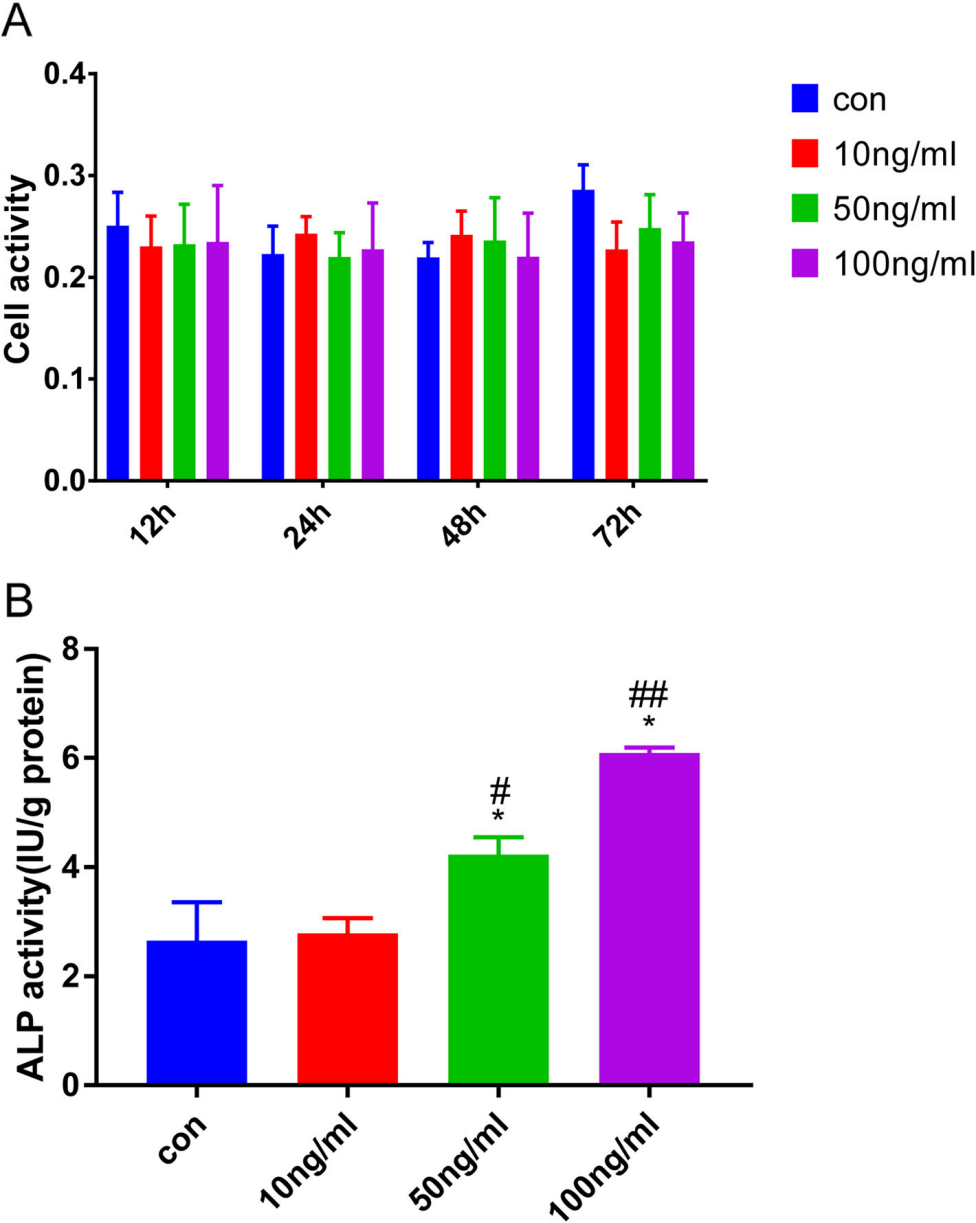
Effects of vaspin on osteoblast proliferation and ALP activity. Vaspin at different concentrations exerted no significantly effects on osteoblast proliferation (**a**). ALP activity quantification was measured on 72 h following treatment of different concentrations of vaspin (**b**). Data are expressed as the mean ± SD. Three independent experiments and repetitions were performed between conditions. ^*^p < 0.05 compared with control group, ^#^*p* < 0.05 compared with 10 ng/mL vaspin group, ^##^*p* < 0.05 compared with 50 ng/mL vaspin group

Next the effect of vaspin on the osteoblast proliferation was determined by CCK-8 activity assay. The results showed that vaspin exerted no significantly cytotoxic effects, even concentrations up to 100 μM. At 12 h, there were no significant differences in osteoblast proliferation in the vaspin groups (10 ng/mL, 50 ng/mL and 100 ng/mL) compared with the control group (*P* > 0.05; Fig. 6a). Similarly, proliferation of the 3 groups had no statistically significant difference compared with the control group at 24 h, 48 h or 72 h (*P* > 0.05; Fig. 6a). The results indicated that vaspin at different concentrations can not affect osteoblast proliferation.

### Effects of vaspin on osteoblast differentiation

To investigate the effects of vaspin on osteoblast differentiation, primary rat osteoblasts were treated with various concentrations of vaspin (10, 50, 100 ng/mL), ALP activity and mRNA levels of the bone differentiation marker genes, including Runx2, Osx and Colla1 were measured. As shown in Fig. 6b, ALP activity assessed at 72 h was enhanced with the increase the vaspin concentrations, and reached a peak at 100 ng/mL vaspin, suggesting that vaspin promoted osteoblastic differentiation in a dose-dependent manner.

**Fig. 7:**
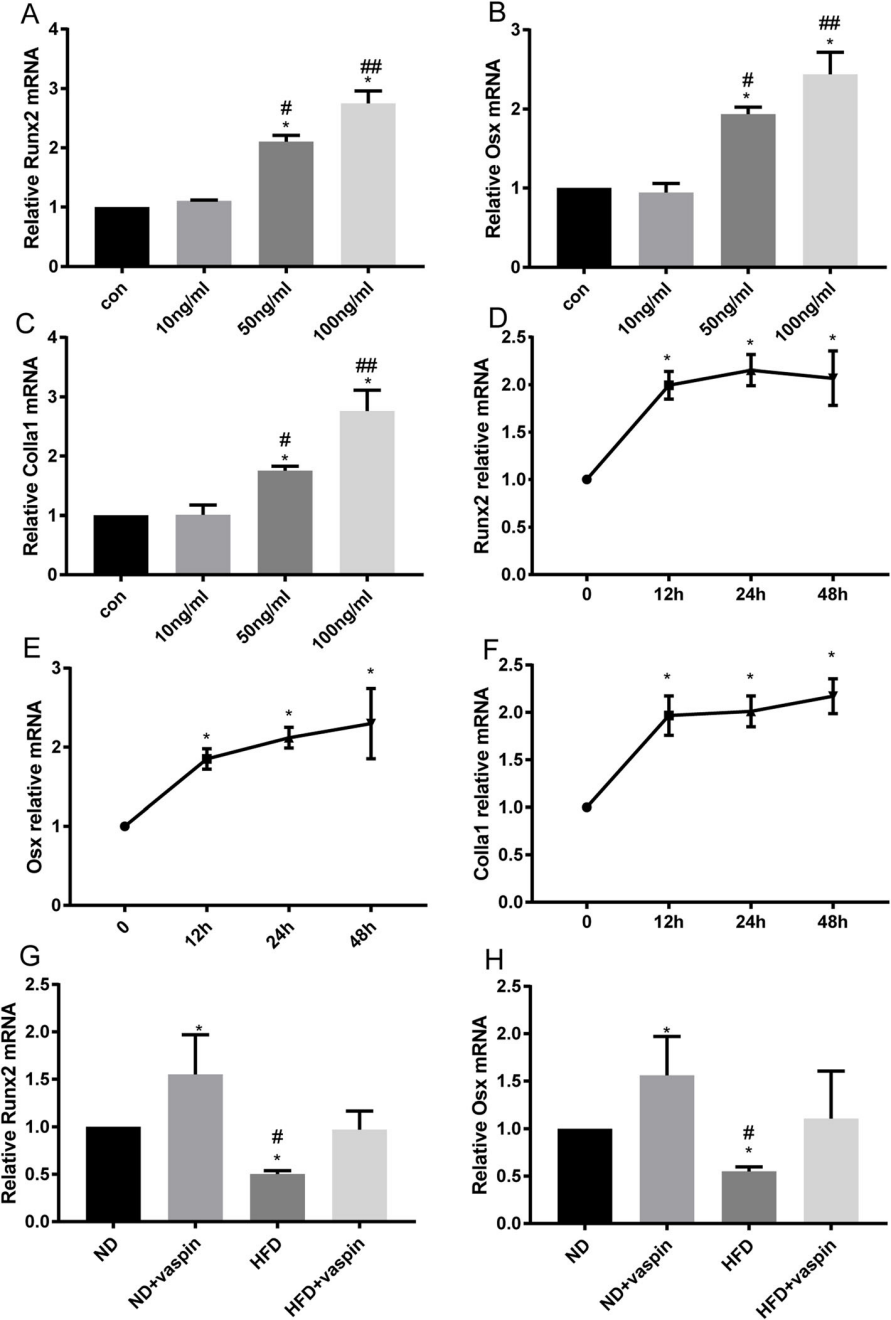
Effects of vaspin on osteoblast differentiation gene expression. After primary rat osteoblasts were exposed to various concentrations of vaspin, the mRNA expression of Runx2 (**a**), Osx (**b**) and Colla1 (**c**) was measured by RT-PCR. The mRNA expression of Runx2 (**d**), Osx (**e**) and Colla1 (**f**) in primary rat osteoblasts treated with 50 ng/ml vaspin was detected at different time points. ^*^p < 0.05 compared with control group, ^#^*p* < 0.05 compared with 10 ng/ml vaspin group, ^##^*p* < 0.05 compared with 50 ng/ml vaspin group. The mRNA expression of Runx2 (**g**) and Osx (**h**) in femurs of four different groups. Data are expressed as the mean ± SD from three independent experiments. ^*^p < 0.05 compared with ND, ^#^*p* < 0.05 compared with HFD + vaspin

In addition, osteoblast differentiation gene expression was investigated following 48 h of vaspin treatment according to previous studies [21, 22, 29]. As presented in Fig. 7, osteoblasts treated with 10 ng/mL vaspin exhibited no significant increase in the Runx2 (Fig. 7a), Osx (Fig. 7b) and Colla1 (Fig. 7c) expression compared with the control, while a significant promotion was first observed in 50 ng/mL vaspin-treated cells and the promotion effects peaked in 100 ng/mL vaspin-treated cells. Our data demonstrated that vaspin promoted osteoblast differentiation in a dose-dependent manner. Then, the time needed for realizing these regulatory effects was detected. A dose of 50 ng/mL vaspin was chosen for the next tests, because this dose caused an obvious response and had no significant cytotoxicity. Primary rat osteoblasts were treated with 50 ng/mL vaspin for different times (0 h, 12 h, 24 h, 48 h). Compared with the control without vaspin stimulation, the mRNA levels of Runx2, Osx and Colla1 were all up-regulated at 12 h, 24 h and 48 h. However, there were no significant differences in the expression of these genes at 12 h (Fig. 7d), 24 h (Fig. 7e) and 48 h (Fig. 7f). The results suggest that vaspin may enhance the differentiation of osteoblasts dose-dependently but not time-dependently.

Further, the osteoblast gene expression was investigated in vivo. It was found that the mRNA levels of Runx2 and Osx were down-regulated in HFD group (Fig. 7g), while the changes in HFD + vaspin group were not significant difference compared to the ND group (Fig. 7h). Furthermore, the ND + vaspin rats demonstrated higher mRNA expressions of Runx2 and Osx than those in ND rats, suggesting that vaspin might promote bone formation in vivo.

### Effects of vaspin on the Smad-Runx2 signaling pathway in primary rat osteoblasts

**Fig. 8:**
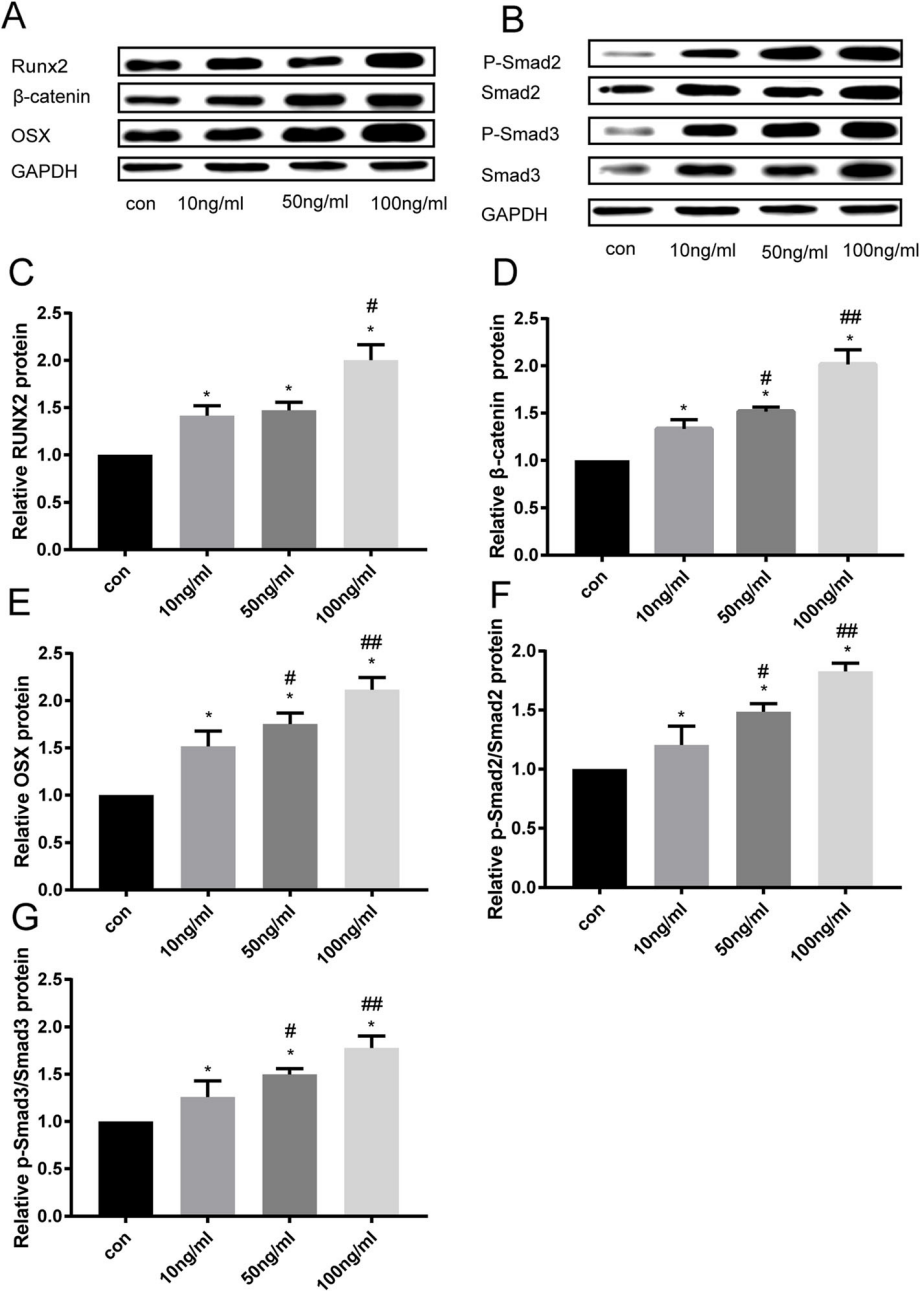
Effects of vaspin on osteogenic protein expression of primary rat osteoblasts. After treated with different concentrations of vaspin protein expression of Runx2, Osx, β-catenin (**a**), Smad2, Smad3, p-Smad2, and p-Smad3 (**b**) in primary rat osteoblasts was detected by Western blot analysis. The relative protein levels were quantified by densitometry and normalized to GAPDH(**c**-**g**). Data are expressed as the mean ± SD from three independent experiments. ^*^p < 0.05 compared with control group, ^#^*p* < 0.05 compared with 10 ng/ml vaspin group, ^##^*p* < 0.05 compared with 50 ng/ml vaspin group

**Fig. 9:**
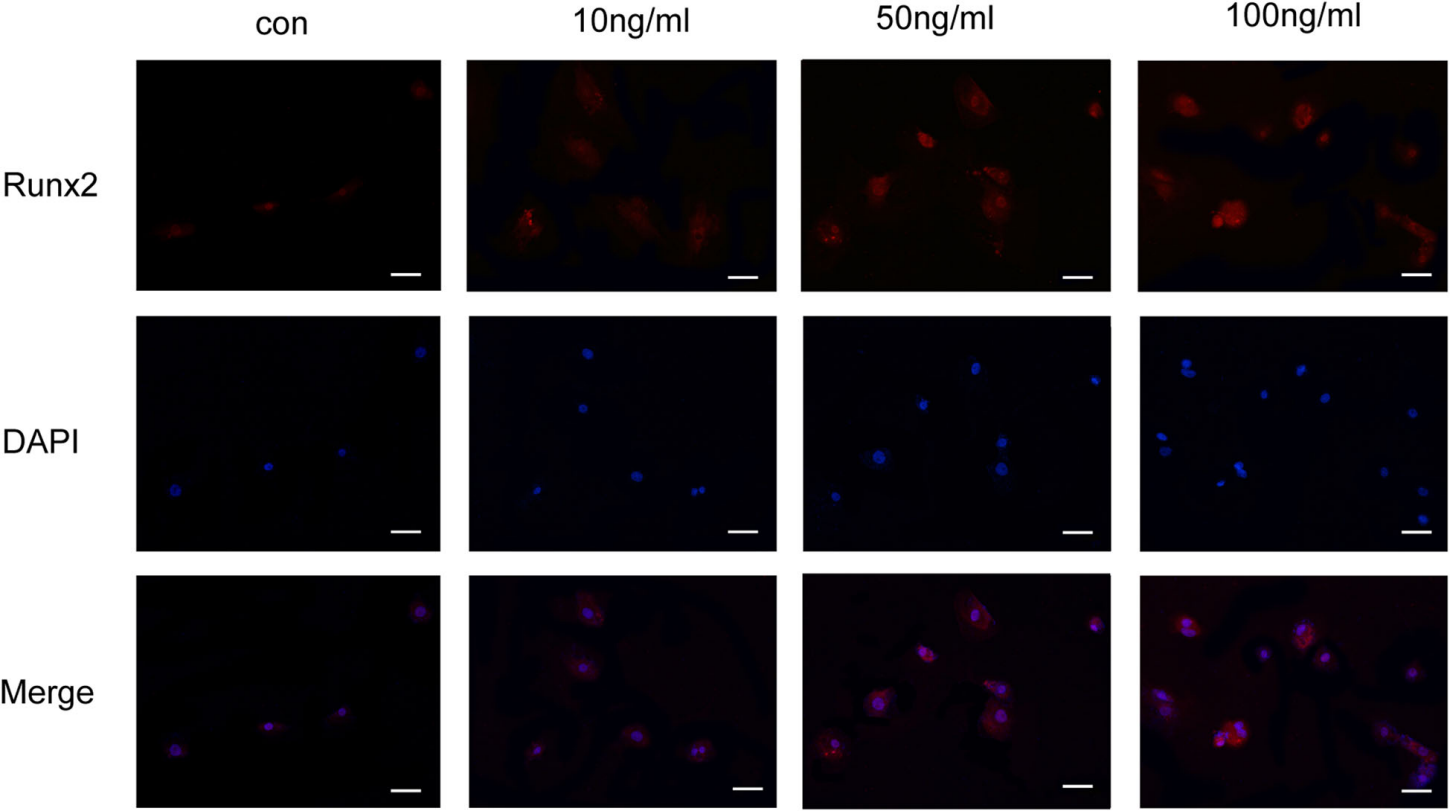
Effects of vaspin on Runx2 signaling pathway. Immunofluorescence staining with anti-Runx2 primary antibodies was used to detect expression of Runx2, original magnification: × 200, bar = 50 μm. The nuclei were stained with DAPI as shown in blue

To further examine whether the Smad-Runx2 signaling pathway was influenced by vaspin, the protein expression of Smad2, Smad3, p-Smad2, p-Smad3, Runx2, Osx and β-catenin in primary rat osteoblasts cells was determined using Western blot analysis after treatment with vaspin (10-100 ng/mL) for 48 h. The results showed that vaspin dose-dependently increased Smad2, Smad3, p-Smad2, p-Smad3, the ratio of p-Smad2/Smad2 and p-Smad3/Smad3, and the Runx2, Osx and β-catein protein levels (Fig. 8). Runx2 was also examined by immunofluorescence. As shown in Fig. 9, treatment with 50 ng/mL vaspin resulted in an increase in the number of Runx2 fluorescent cells and peaked at 100 ng/ml compared with the control cells. It was indicated that vaspin prompted phosphorylation of smad2/3 and the nuclear accumulation of runx2, resulting in the activation of the Smad-Runx2 signaling pathway, therefore promoted the osteoblast differentiation of rat osteoblasts cells.

## Discussion

In the present study, we investigated the effects and mechanisms of vaspin in bone metabolism in vivo and in vitro. The major finding of this study was that vaspin supplement antagonized the bone loss, promoted osteogenic differentiation and preserved normal bone mass in HFD-induced obese rats and primary rat OBs.

Our study showed that in HFD group, there was a significant increase in body weight, but these effects were improved after treatment with vaspin. The effects of vaspin on weight and appetite were controversial in previous in vivo animal experiments. Brunetti et al. [19] confirmed that compared to vehicle, vaspin injection into the arcuate nucleus of the hypothalamus(1μg/kg, once, *n* = 9) significantly decreased food intake in the 24 h following treatment. Kloting et al. [20] found that both peripheral (1 mg/kg, intraperitoneal injection daily on experimental days 1, 3 and 4, *n* = 6) and central vaspin administration (1μg/kg, intracerebroventricular injection for once, *n* = 8) acutely decrease food intake in female obese db/db and lean C57BL/6 mice, they considered that the central administration of vaspin to rats subdues appetite, thereby reducing body weight. Kameshima et al. found that body weight of spontaneously hypertensive rats (SHR) was significantly lower than that of control group, of note, vaspin treatment (1 μg/kg/day, intraperitoneally, *n* = 7) for 4 weeks seemed to recover the body weight of SHR [30]. Sakamoto et al. [31] examined the effects of vaspin (1μg/kg/day, 14 days, *n* = 4) treatment on MCT-induced rise in pulmonary arterial hypertension (PA) pressure and RV to body weight ratio (RV/BW), and demonstrated that vaspin significantly attenuate the rise in PA pressure but have no effect on the body weight. While Liu et al. [32] showed that a downward trend in body weight could be seen in HFD- fed rats treated with vaspin (320 ng/kg/day, intraperitoneally, *n* = 10) for 8 weeks except for significantly decreasing fasting blood glucose and fasting insulin concentrations. However, in this study, no statistically significant.in food intake could be seen in rats treated with vaspin. We considered that the difference between this study and others could be partly due to the different ways of vaspin administration, different doses, different time of treatment and different vaspin serum concentrations between the different models. As vaspin was injected intraperitoneally in our study, and the level of vaspin that reached the central nervous system via circulation was too low to inhibit feeding behavior. However, serum vaspin concentration in the rodents remains unknown. The dose (1 μg/kg/day, intraperitoneally) was used in the present study according to the previous in vivo animal experiments, as this vaspin concentration is considered within pathophysiologically reasonable range because blood vaspin concentration is estimated ~ 14.3–17.2 ng/ml if the circulating blood volume is approximately 64 ml/kg in the rats [31, 33]. Further research is needed in terms of vaspin serum concentrations between the different models. Furthermore, the number of rats in the experiment was limited, so this difference may become statistically significant as the sample size increased [34].

Obesity can cause insulin resistance and decrease the body’s metabolic rate. Consistent with the clinical features of obese patients, our research showed that the VO_2_, VCO_2_, RER, heat production and physical activity were significantly reduced in the HFD rats as compared to the ND rats, these results suggested that the whole body metabolism was significantly reduced in the HFD rats. Nevertheless, vaspin can improve the VO_2_, VCO_2_, RER, heat production and physical activity caused by obesity, increase energy expenditure and body’s metabolic rate. Previous studies confirm that vaspin can improve glucose metabolism and insulin sensitivity in obesity, attenuate adipose tissue inflammation, taken together, partially ameliorate the adverse effects of DIO. Thereby we speculate the mechanisms of vaspin on decreasing the body weight are likely to closely related to the above reasons [34]. Although the mechanisms underlying the effects of vaspin on energy expenditure remain not well understood, our data suggest a previously unrecognized role of vaspin in losing weight.

Bone turnover markers can be used to monitor the effects of osteoporosis treatment. The International Osteoporosis Foundation has proposed that the reference marker for bone formation should be serum PINP (produced by osteoblasts during bone formation), and serum CTX for bone resorption (released by osteoclasts during bone resorption) [35]. We observed a significant decrease in plasma PINP and ALP levels and a significant increase in plasma CTX level in HFD group. However, the administration of vaspin can reversed this phenomenon, indicating that vaspin inhibits bone resorption and promotes bone formation. Biomechanical strength is a high-profile indicator reflecting the fragility and fracture risk of bone, which is closely related to bone microstructure [36]. To explore the role of vaspin in bone metabolism, we conducted a bone biomechanical analysis of the right femurs. We found that the HFD group had significantly reduced bone strength relative to the ND group, and the administration of vaspin restored the bone strength to a normal level. These findings clearly showed that vaspin played a role in the maintenance of bone strength.

Trabecular bone microarchitecture is generally considered an ideal index for predicting bone loss and bone structure deterioration [37]. Our findings also showed that obesity induced by an HFD is detrimental to trabecular bone of femur in rats. These structural changes are associated with a decrease in bone formation and an increase in bone resorption in HFD-fed rats [38]. In accordance with the previous studies [37, 39], this study suggested that the rats model of trabecular bone loss can be established by DIO. Of note, DIO failed to induce trabecular bone loss in the treatment of vaspin, indicating that vaspin played a critical role in the pathological process of obesity-related trabecular bone loss. However, neither the HFD diet nor the vaspin administration showed effects on cortical parameters. The finding of trabecular bone rather than cortical bone affected by the HFD and vaspin is not surprising. In general, trabecular bone is more responsive than cortical bone to diet or drug treatments, physiological status, or aging because the trabecular bone has a higher remodeling activity than cortical bone due to the larger surface to volume ratio [37]. Other possible explanations are that: 1) midshaft cortical bone might not have enough time to adapt the way of macromodeling; 2) bone geometry at the midshaft did not change due to biochemical and/or endocrinological constraints imposed by the fat:lean ratio; or 3) geometry changes did not reflect the additional weight of obese rats [8]. We also investigated the effects of vaspin on the osteoblastogenesis of primary rat osteoblasts. The CCK8 results indicated that vaspin could not affect osteoblast proliferation. RT-PCR results revealed that HFD decreased mRNA expressions of Runx2, Osx and Colla1. On the other hand, higher mRNA expressions of Runx2, Osx and Colla1 were found after treatment of vaspin on the same diet, indicating that vaspin may have the potential to activate osteoblastogenesis in primary mouse osteoblasts.

Osteoblast differentiation is the key to bone formation, in which bone morphogenetic TGF-β/Smad signaling pathway is involved [40]. The previous studies revealed that Smad2/3 played an important role in the regulation of bone formation [41], and the expressions of Smad2/3 and p-Smad2/3 were elevated during cementoblast differentiation and mineralization [42]. Typically, the activation of the TGF-β/Smad signaling pathway mediates the phosphorylation of Smad2/3, which dimerizes with Smad4 and translocates to the nucleus, resulting in downstream gene transcription to direct cell differentiation [43]. Runx2 and Osx are important transcription factors for osteoblast differentiation and bone formation [44]. Moreover, Runx2 directly interacts with the promoter regions of key osteoblast specific genes, including ALP, osteocalcin (OCN) and β-catenin. In the present study, we discovered that vaspin significantly upregulated the expression of Smad2, Smad3, p-Smad2, p-Smad3 and the ratio of p-Smad2/Smad2 and p-Smad3/Smad3, thereby resulting in an significant increase of Runx2 and Osx expression. Protein expression of Runx2 was analyzed by immunofluorescence staining, and the result was consistent with the result of Western blot. The results showed that Runx2 was activated by vaspin at both mRNA and protein levels, supporting that vaspin promoted osteogenic differentiation via the Smad-Runx2 signaling pathway.

To the best of our knowledge, this is the first study to investigate the effects of vaspin on bone in vivo, and demonstrates that vaspin antagonizes the bone loss induced by HFD, and promotes osteoblastic differentiation by activating the Smad-Runx2 signaling pathway in vitro. However, it is important to mention that the present study has several limitations. First, we should use palmitic acid to study the direct effect of fatty acid on osteoblastogenesis in vitro. Second, we did not use the inhibitor of the smad2/3 signaling pathway. Third, we did not investigate the effect of vaspin on the osteoclasts. Therefore, further studies are needed to identify the mechanism of vaspin and the bone metabolism.

## Conclusion

In conclusion, this study identified that vaspin protected the HFD-induced trabecular bone loss in vivo. Moreover, in vitro studies demonstratesd that vaspin promoted osteoblastic differentiation by activating the Smad-Runx2 signaling pathway, which subsequently activated the downstream smad2/3-targeted gene transcription for osteogenesis. Our findings provided new insights for exploring the mechanism of vaspin functioning on bone formation. Thus, vaspin might be used as the therapeutic agent for treating obesity and HFD-induced bone loss.

## Data Availability

All data generated or analysed during this study are included in this published article or are available from the corresponding author on reasonable request.

## Abbreviations

ALP: Alkaline phosphatase
AUC: Area under the curve
BMD: Bone mineral density
Ca: Calcium
Colla1: Collegen alpha1
Ct. BV/TV: Cortical bone volume/total volume
Ct. Th: Cortical bone thickness
Ct.vBMD: Cortical volume bone mineral density
CTX: C-telopeptide of type I collagen
DIO: HFD-induced obesity
H: Heat production
HDL: High-density lipoprotein cholesterol
HFD: High fat diet
IL-1: Interleukin-1
IL-6: Interleukin-6
LDL: Low-density lipoprotein cholesterol
Micro-CT: Microcomputed tomography
ND: Normal diet
OBs: rat primary osteoblasts
OCN: Osteocalcin
OLETF: Otsuka Long-Evans Tokushima Fatty
OPG: Osteoprotegerin
Osx: Osterix
P: inorganic phosphorus
PINP: Procollagen I N-terminal peptide
PPAR-γ: Peroxisome proliferator-activated receptor gamma
RER: Respiratory exchange ratio
Runx2: Runt-related transcription factor 2
SD: Sprague-Dawley
serpin: Serine protease inhibitor
SMI: the structure model index
Tb.BV/TV: Trabecular bone volume/total volume
Tb.N: Trabecular number
Tb.Sp: Trabecular separation
Tb.Th: Trabecular thickness
Tb.vBMD: Trabecular volume bone mineral density
TC: Total cholesterol
TG: Triacylglycerol
TGF-β: Transforming growth factor-β
TNF-α: Tumor necrosis factor α
Vaspin: Visceral adipose tissue-derived serine protease inhibitor
VCO2: production of carbon dioxide
VO2: Volume of oxygen consumed
